# Consumer Acceptance and Quality Parameters of the Commercial Olive Oils Manufactured with Cultivars Grown in Galicia (NW Spain)

**DOI:** 10.3390/foods9040427

**Published:** 2020-04-03

**Authors:** Sol Zamuz, Laura Purriños, Igor Tomasevic, Rubén Domínguez, Mladen Brnčić, Francisco J. Barba, José M. Lorenzo

**Affiliations:** 1 Centro Tecnológico de la Carne de Galicia, Parque Tecnológico de Galicia, 32900 Ourense, Spain; solzamuz@ceteca.net (S.Z.); laurapurrinos@ceteca.net (L.P.); rubendominguez@ceteca.net (R.D.); 2 Faculty of Agriculture, Department of Animal Source Food Technology, University of Belgrade, Nemanjina 6, 11080 Belgrade, Serbia; tbigor@agrif.bg.ac.rs; 3 University of Zagreb, Faculty of Food Technology and Biotechnology, Pierottijeva ulica 6, 10000 Zagreb, Croatia; mbrncic@pbf.hr; 4 Universitat de València, Faculty of Pharmacy, Preventive Medicine and Public Health, Food Science, Toxicology and Forensic Medicine Department, Nutrition and Food Science Area, Avda.Vicent Andrés Estellés, s/n, 46100 Burjassot, València, Spain; Francisco.Barba@uv.es

**Keywords:** Brava, Mansa, EVOO, quality parameters, fatty acids, sensory acceptance, volatile compounds

## Abstract

Mansa and Brava are olive autochthonous cultivars from Galicia, a new olive-growing zone from NW Spanish, from which high-quality extra virgin olive oils (EVOOs) are obtained. The oils obtained as by co-crushing Mansa and Brava olives in different proportions as by blending with others olives cultivars have different composition that influence in their sensory quality. The consumer acceptance of commercial oils elaborated with Local Galician cultivars was evaluated and a quality-mapping of olive oils was created. It was found that the both Local oils had good physical-chemical quality parameters. From sensory analysis viewpoint, Local-MB oils presented the highest intensity values for color, odor, taste, and flavor, and the consumers had a higher acceptance and preference by Picual, Local-MBPA (60% Mansa and Brava, 25% Picual, and 15% Arbequina and Local-MB (60% Mansa and 40% Brava) oils. A quality-mapping of olive oils indicate that attributes better scored from the consumer are high intensity for color, odor, taste and flavor, and pungent and floral series, and bitter is rejected by them.

## 1. Introduction

Spain ranks first in olive grove area and the main olive-growing zone in terms of production is Andalucía (South Spain) due to the warm and dry climate [1,2]. Although the climate in Galicia (NW Spain) is typically defined as Atlantic climate, there are different areas with Mediterranean climate where the best climatic conditions for olive growing are given [3,4]. Galicia has gradually emerged as a new Spanish olive-growing zone [5] and though Arbequina and Picual varieties predominate in plantations, Mansa and Brava are olive autochthonous cultivars (known by producers as Local) from which high-quality extra virgin olive oils (EVOOs) are obtained. The current trend of the olive oil market is the production of EVOOs with specificity of origin and particular and differentiated sensory, nutritional, and health characteristics [6].

The EVOO is highly appreciated by consumers for its nutritional properties and its healthy effects [7]. Several studies shown that oils obtained as by co-crushing Mansa and Brava olives in different proportions (usual practice adopted by elaboration of these oils) as by blending these Local olives with Picual and/or Arbequina olives have differentiated aromatic, fat, and phenolic composition which results in different organoleptic attributes and sensory characteristics influencing the sensory quality [8,9,10,11].

The sensory concept is a multidimensional concept that include both sensory evaluations performed by trained experts panel coupled with acceptance and preferences of the consumers [12]. The two quality and commercial categories (extra virgin olive oil and virgin olive oil) are established by taste panel according to the sensory analysis carried out following official method supported by International Olive Council (IOC) [13]. This evaluation takes into account three positive attributes for olive oils (fruity, bitter and pungent) and absence of five main defects (rancid, musty, winey, metallic, and fusty). However, the rejection of bitterness and pungency is a natural reaction and these attributes are frequently rejected by consumers, who related this sensory characteristic with poisonous or toxic substances [14]. Thus, in contrast with trained panelist, most of consumers do not relate these characteristics as positive sensorial attributes of olive oil [15]. There are fewer avenues for consumers to become acquainted with expert recommendations which have influence in buying decisions. Thus, acceptance and preference evaluations are very important in the oil industry and are being considered as analytical tools to evaluate marketing acceptability of new EVOOs.

In this context, the main objective of this study was to evaluate the consumer acceptance of commercial EVOOs elaborated with Local Galician cultivars and experimental monovarietal oil elaborated with Mansa cultivar. A second goal was to compare the consumer acceptance of EVOOs elaborated with autochthonous Galician cultivars with the consumer acceptance of others commercial monovarietal oils elaborated with Picual and Arbequina cultivars which are highly appreciated by consumers. Finally, quality-related parameters were determined in all studied oils to establish the relations between them and the acceptance results and to create the quality-mapping of olive oils from the consumer viewpoints.

## 2. Materials and Methods

### 2.1. Oil Samples

Three different Local oils elaborated with Galician olives were studied. Two commercial oils, labelled and marketed as EVOOs: 1) elaborated exclusively with Local Galician cultivars (60% Mansa and 40% Brava, named Local-MB) and 2) elaborated by blending Local Galician cultivars with Picual and Arbequina (30% Mansa, 30% Brava, 25% Picual, and 15% Arbequina; named Local-MBPA). The third oil that was evaluated was one experimental oil batch elaborated with 100% Mansa cultivar (Man). Olives were grown and harvested in two crop seasons (2017 and 2018) in Quiroga, an area located in the in the valley of River Sil (Lugo province, Galicia, NW Spain). Local-MB, Local-MBPA, and Man batches were elaborated following a cold-pressed procedure typically used by local producers and marketed by Ouro de Quiroga, S.L. (Quiroga, Spain) that is used to provide the oil samples. Commercial EVOOs elaborated with 100% Picual (Pic) and with 100% Arbequina (Arb) were purchased at a local supermarket. Picual EVOO belongs to DOP Sierra de Segura (Jaén, Spain) and Arbequina EVOO belongs to DOP Les Garrigues (Lleida, Spain). Olive oils samples were kept under dark conditions to protect them from light until they were analyzed.

### 2.2. Quality-Related Parameters and Fatty Acids Composition in Oil

Free acidity (% oleic acid) and peroxides (meq O_2_/kg oil) were quality-related physicochemical parameters determined by analytical methods established in European Commission Regulation [16,17]. Fatty acids composition was determined according to the method described by Barros et al. [18]. Briefly, 20 milligrams of olive oil were transesterified using sodium methoxide and methanolic solution of sulfuric acid. Then, fatty acid methyl esters were separated using hexane. The separation and quantification was carried out using a gas chromatograph (GC-Agilent 7890B; Agilent Technologies Spain, S.L., Madrid, Spain) equipped with a flame ionization detector, following the chromatographic conditions described by Barros et al. [18]. The fatty acids amounts were calculated based on the internal standard technique, using nonadecanoic acid as internal standard (I.S. C19:0; 300 ppm). The results of fatty acid profile were expressed as % of total fatty acids, while the sums of saturated, monounsaturated, and polyunsaturated were expressed as mg/g of oil.

### 2.3. Lipid Oxidation Parameters

The anisidine and TOTOX values were also determined to measure the oil oxidation. Anisidine value (AV) was determined using iso-octane following IUPAC method [19]. TOTOX value indicate the overall oxidation state [20] of the oil and it was calculated according to the formula [21]:

(1) TV=AV+2 PV

### 2.4. Volatile Compounds

The extraction of the volatile compounds was performed using solid-phase microextraction (SPME) with an autosampler Pal RTC-120. The oil sample (1 g) was weighed in a 20-mL vial (Agilent Technologies, Santa Clara, CA, USA) and subsequently screw-capped with a laminated Teflon-rubber disc. Then, this vial was conditioning at 37 °C during 15 min and the extraction process was carried out at the same temperature during 30 min. At that point, volatile compounds adsorbed into SPME fiber were desorbed in gas chromatograph inlet and separated, identified, and quantified in a gas chromatograph 7890B GC-System (Agilent Technologies, Santa Clara, CA, USA) equipped with a mass selective detector 5977B MSD (Agilent Technologies), following the method described by Domínguez et al. [22]. The chromatogram integration was done with Agile2 algorithm (MassHunter Quantitative Analysis B.07.01), while peak detection was done with deconvolution. Compounds were identified by comparing their mass spectra with those contained in the NIST14 library (National Institute of Standards and Technology, Gaithersburg). The compounds were considered as correctly identified when their spectra presented a library match factor >85%. After integration, peak detection and identification of each compound, the extraction ion chromatogram (EIC) from the quantifier ion was obtained from each peak. The final results were expressed as area units of the EIC × 10^4^ per gram of oil (AU-EIC × 10^4^/g of oil).

### 2.5. Sensory Evaluation

The sensory tests for the evaluation of olive oils were conducted in the sensory laboratory of the Meat Technology Centre of Galicia (Ourense, Spain) and held in closed individual booths according to Regulation [23], under white light. Samples were analyzed in two sessions (1 per crop season 2017 and other 2018) and five samples (1 per batch) were offered to the taster coded with random 3-digit number. Water and green apple were used to clean the palate and remove residual flavors. A total of 70 consumers (42 females and 28 males aged from 25–40 years) took part in the study, and they were informed about the objectives of the study and the instructions to complete tests by a trained interviewer before to begin. Consumers were select on the basis of their availability for the evaluation, interest to participate in the research and moderate preference towards olive oils.

To determinate how the consumers liked or dislike the olive oil samples, the acceptance test was carried out using a hedonic scale structured in 7-points (1 = dislike very much and 7 = like very much) according to Lago et al. [24] for evaluated the overall liking of each oil. Additionally, the preference test [25] was conducted together with acceptance test, using a structured 5-point scale (1 = less favorite and 5 = most favorite).

Previously at sensory analysis, consumers were asked which sensory attributes were considered by them to evaluate overall liking of olive oils. The sensorial attributes considered were color, odor, taste, and flavor. Thus, either consumer also evaluated the intensity of these sensorial attributes, using a lineal structured scale from 0 (sensation not perceived) to 10 (maximum sensation) following a randomized complete equilibrated block design.

### 2.6. Statistical Analysis

The differences in quality-related physicochemical parameters, fatty acid profile, and lipid oxidation parameters among different olive oils samples were examined using a one-way ANOVA and Duncan’s test was used to determine significant differences. Statistical significance was given at *P* ≤ 0.05 after post hoc comparison. Friedman two-way ANOVA, assuming product and taster as independent factor, was used to analyze the obtained data of preference test. When a significant effect (*P* ≤ 0.05) was found, LSD was used as a multiple comparison test. Finally, external preference mapping (PREFMAP) was created to relate consumer acceptance, sensorial attributes, physicochemical parameters, and fatty acid and volatile composition of either olive oil samples to stablish a quality-mapping of olive oils from the consumer viewpoints [12,26]. XLSTAT for Windows version 2018 (Addinsoft, Paris, France) was used to analyze data.

## 3. Results and Discussion

### 3.1. Quality-Related Indices in Olive Oils: Physicochemical and Fatty Acids Composition

The results obtained for the physicochemical parameters and fatty acids considered as quality-related indices of five studied olive oils allow to classify olive oils within the different categories established by the European Commission and their values are regulated by European law [16,17]. As one would expect, commercial olive oils presented values of free acidity (% oleic acid) and peroxides (meq O_2_/kg) content was lower that established limit (0.8% and 20 meq O_2_/kg, respectively) (data not showed). The obtained values for free acidity in *Local*-MBPA, *Local*-MB and Mansa oils were similar to those found in other oils elaborated with *Mansa* and *Brava* cultivars [9,10] and peroxide values were lower. Experimental *Mansa* oil showed values of 0.31% oleic acid and meq 8.41 O_2_/kg and therefore also could be considered as EVOOs according to considered physicochemical parameters.

In the same way, the values of fatty acids of commercial EVOOs were lower that established limit by European law (Table 1).

The results obtained to oil elaborated with 100% *Mansa* cultivar exceed the limit ranked for C18:3n-3 (1.03%), so experimental *Mansa* oil cannot be considered EVOO. The most abundant fatty acids in all olive oils have been C18:1n-9, C16:0, C18:2n-6, and C18:0, as can be observed in literature [9,10,27]. According to the statistical analysis, *Picual* oil showed the highest values (*P* ≤ 0.05) for C18:1n-9 and C18:0 with values of 77.72% and 2.77%, respectively, and the lowest (*P* ≤ 0.05) for C16:0 and C18:2n-6 with values of 11.29% and 3.74%, respectively. *Arbequina* oil showed the highest values (13.53%) for C16:0. The highest values for C18:2n-6 were found in *Mansa* oil (11.75%), which also presented the lowest values (67.07% and 1.94%) for C18:1n-9 and C18:0, respectively. The C18:1n-9 and C18:0 content of *Local*-MBPA and *Local*-MB oils were similar to *Picual* oil and the C18:2n-6 and C16:0 were similar to *Arbequina* oil, except the C14:0 content of *Local*-MB oil that was similar to *Picual* oil.

These results agreed with the others authors who observed that *Picual* oils have high C18:1n-9 content and low C18:2n-6 contents. *Arbequina* oil shows an opposite composition at *Picual* oils [28] and *Local* oils were intermediate between mentioned varieties, similar to others realized works [9]. *Mansa* oils presented a surprising high content of C18:3n-3, near 1%, which is characteristic of some olive oils from Moroccan [29]. On the other hand, the obtained results for fatty acids in *Mansa* oils did not agree with they showed by Reboredo-Rodríguez et al. [1], being more similar to presented in *Brava* oils.

**Figure 1:**
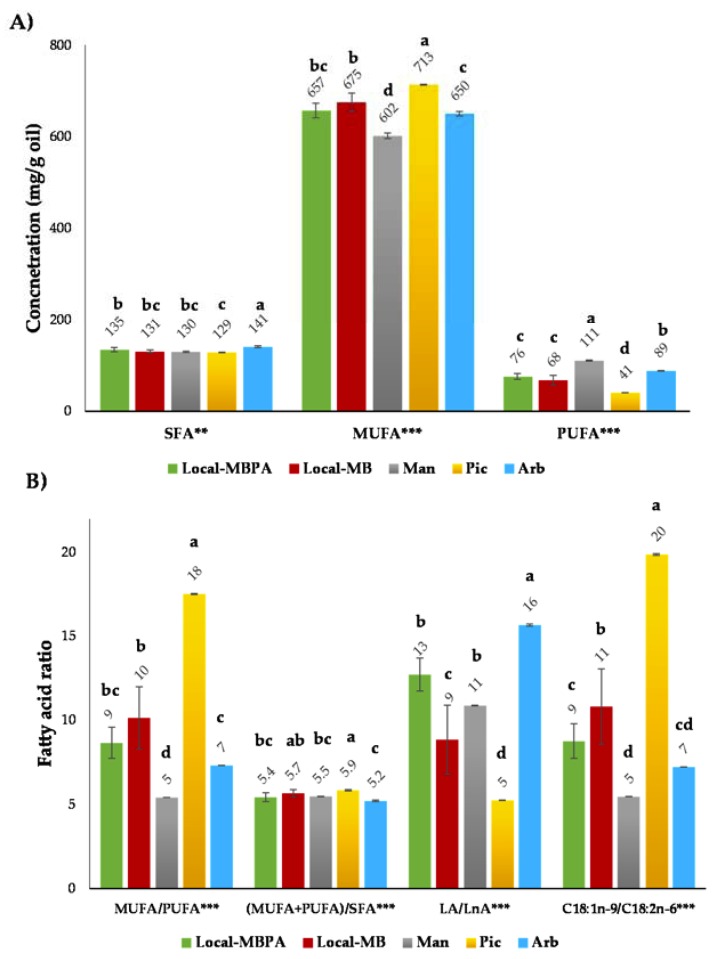
Fatty acid composition (**A**) and fatty acid ratio (**B**) of the virgin olive oils (VOOs). ** (*P* ≤ 0.01); *** (*P* ≤ 0.001). ^a–d^ Mean values in the same row with different letters indicate significant differences (*P* < 0.05). Local-MBPA (60% Mansa and Brava, 25% Picual, and 15% Arbequina); Local-MB (60% Mansa and 40% Brava); Man (100% Mansa cultivar); Pic (100% Picual cultivar); Arb (100% Arbequina cultivar).

In addition, saturated fatty acids (SFA), monounsaturated fatty acids (MUFA), polyunsaturated fatty acids (PUFA), and four ratios between them (MUFA/PUFA, (MUFA+PUFA)/SFA, LA/LnA, and C18:1n-9/C18:2n-6) were estimated and significant differences (*P* ≤ 0.05) were observed (Figure 1A,B).

MUFA were the predominant fatty acid group in all olive oils and these compounds are important due to its nutritional value and oxidative stability [8]. *Picual* oils showed the highest concentration of MUFA (713 mg/g) following *Local* oils (675 and 657 mg/g in *Local*-MB and *Local*-MBPA, respectively), and *Mansa* oils with the lowest content (602 mg/g). Significant differences (*P* ≤ 0.001) were observed between samples for MUFA. The second fatty acid group were SFA with values of 141 mg/g in *Arbequina* oils and 129 mg/g in *Picual* oils and significant differences (*P* ≤ 0.01) between studied oils. SFA of *Local* and *Mansa* oils were intermediate between the above-mentioned oils. A high content of SFA produce a fatty sensation effect in the mouth due to lead an increase of viscosity and persistence [10]. Finally, PUFA also presented significant differences (*P* ≤ 0.001) between oils; *Mansa* oil showed the highest values (111 mg/g) and *Picual* oils the lowest. PUFA are used as indicators of oxidation due to double bonds in the hydrocarbon chain [21] and they are related to healthy benefits.

In this sense, there are various fatty acid indices that are good quality and stability indicators of olive oils. All calculated fatty acid indices showed significant differences (*P*≤0.001) between oils (Figure 1B). Both MUFA/PUFA and C18:1n-9/C18:2n-6 ratios are important parameters and high values favor the resistance to oxidation [10]. *Picual* following of *Local*-MB and *Local*-MBPA samples were the oils with the highest values for MUFA/PUFA and C18:1n-9/C18:2n-6. The ratio (MUFA+PUFA)/SFA was also higher in *Picual* and *Local*-MB oils which reduces the fatty sensation in the mouth, favoring organoleptic characteristics [8]. Finally, LA/LnA [C18:2n-6 (LA) and C18:3n-3 (LnA) are essential PUFAs] ratio was related with healthy benefits and a lower value is more desirable in reducing risk of the chronic diseases [8]; *Picual* and *Local*-MB oils were again the samples with better values. In view of the obtained results, *Picual* and *Local*-MB oils showed the best quality parameters.

### 3.2. Lipid Oxidation Parameters in Olive Oils

**Figure 2:**
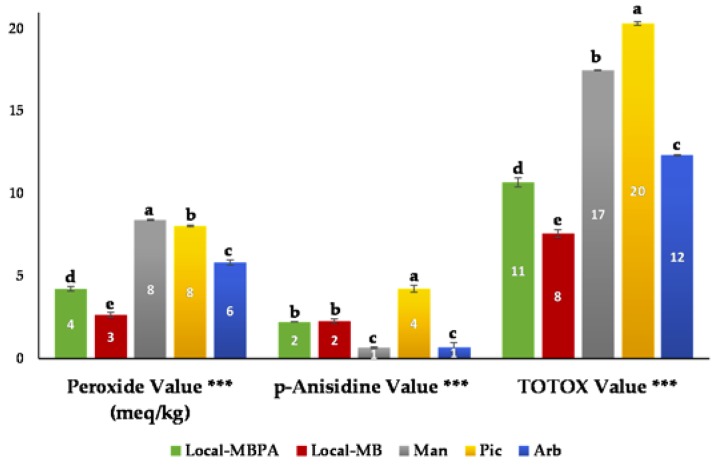
Lipid oxidation parameters of the VOOs. *** (*P* ≤ 0.001). ^a–d^ Mean values in the same row with different letters indicate significant differences (*P* < 0.05). Local-MBPA (60% Mansa and Brava, 25% Picual, and 15% Arbequina); Local-MB (60% Mansa and 40% Brava); Man (100% Mansa cultivar); Pic (100% Picual cultivar); Arb (100% Arbequina cultivar).

Oxidation is a complex series of reactions that could produce rancidity and off flavors and smells, degrading the quality of oils and a series of breakdown products are produced [30,31]. Due to fact, lipid oxidation parameters are also measured in olive oils (Figure 2). The oxidation measure involves methods to evaluate primary and secondary breakdown products.

Peroxide values, besides allow classifying olive oils within the commercial categories established by the European Commission, are also a good primary oxidation indicator. In general, low peroxide values indicate better quality of the oils. The lowest peroxide values were found in *Local*-MB and *Local*-MBPA oils, showed significant differences (*P* ≤ 0.001). The values were lower than 10 meq O_2_/kg, the limited values considered as acceptable for sensory attributes [30].

p-Anisidine value measure the formation of secondary breakdown products and in the same way as peroxide value, low values indicate better quality. In this case, the lowest values were found in *Arbequina* and *Mansa* oil and presented significant differences (*P* ≤ 0.001). TOTOX value was the last measured quality indices and indicates an oil’s overall oxidation state, and again low values indicate better quality. *Local*-MB and *Local*-MBPA were samples oils with the lowest values and also presented significant differences (*P* ≤ 0.001). As can be seen from these results of lipid oxidation parameters, both *Local* oils showed a good oxidative stability that involves a good quality. The different results observed in these oxidation indices could be due to peroxide values, which is a good way to measure primary oxidation products, and p-anisidine values is a good to measure secondary oxidation products.

### 3.3. Volatile Compounds in Olive Oils

Volatile compounds together with phenolic compounds and fatty acids are responsible of sensory perceptions (mainly aroma and tactile mouth sensations) and influencing olive oil quality [9]. Both aroma and mouth sensations cannot be ascribed to a single compound but rather to a mixture and a single compound can be involved in different aromas and mouth sensations. Thirty-three volatile compounds (Table 2) were separated, identified, and grouped into six odorant series (fruity, floral, grass, wood, fatty, and spicy) and three mouth sensations (sweet, bitter, and pungent).

**Figure 3:**
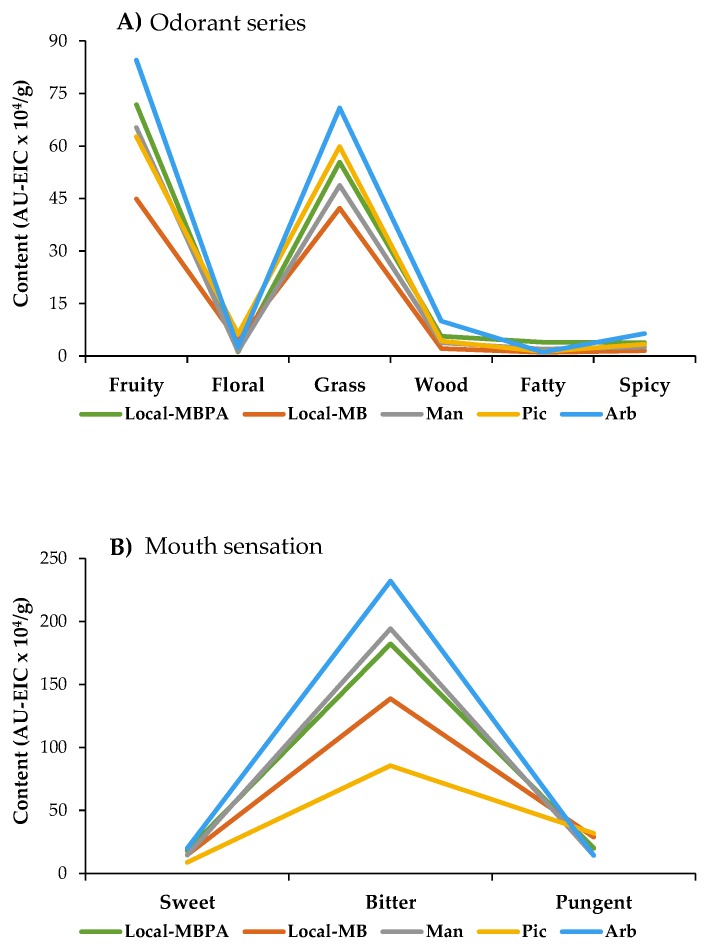
Comparison of odorant series (**A**) and mouth sensation (**B**) in virgin olive oils. Local-MBPA (60% Mansa and Brava, 25% Picual, and 15% Arbequina); Local-MB (60% Mansa and 40% Brava); Man (100% Mansa cultivar); Pic (100% Picual cultivar); Arb (100% Arbequina cultivar).

Figure 3 shows the contribution of each odorant series and mouth sensation to the sensorial profile of studied olive oils.

As can be observed, *Local*-MBPA oils could be characterized by fruity, fatty, and sweet series; *Local*-MB by floral and pungent series; *Mansa* oils by bitter series; *Picual* oils by floral and pungent series; and *Arbequina* oils by fruity, floral, grass, wood, spicy, sweet, and bitter series. *Local*-MBPA oils registered higher volatile compounds content than *Local*-MB oils, probably due to enrichment given by *Arbequina* cultivar. Studies show that it is possible to discriminate oils according to the growing region and the cultivar of the olives on the basis of volatile profile [15].

### 3.4. Sensory Attributes and Consumer Acceptance of Olive Oils

**Figure 4:**
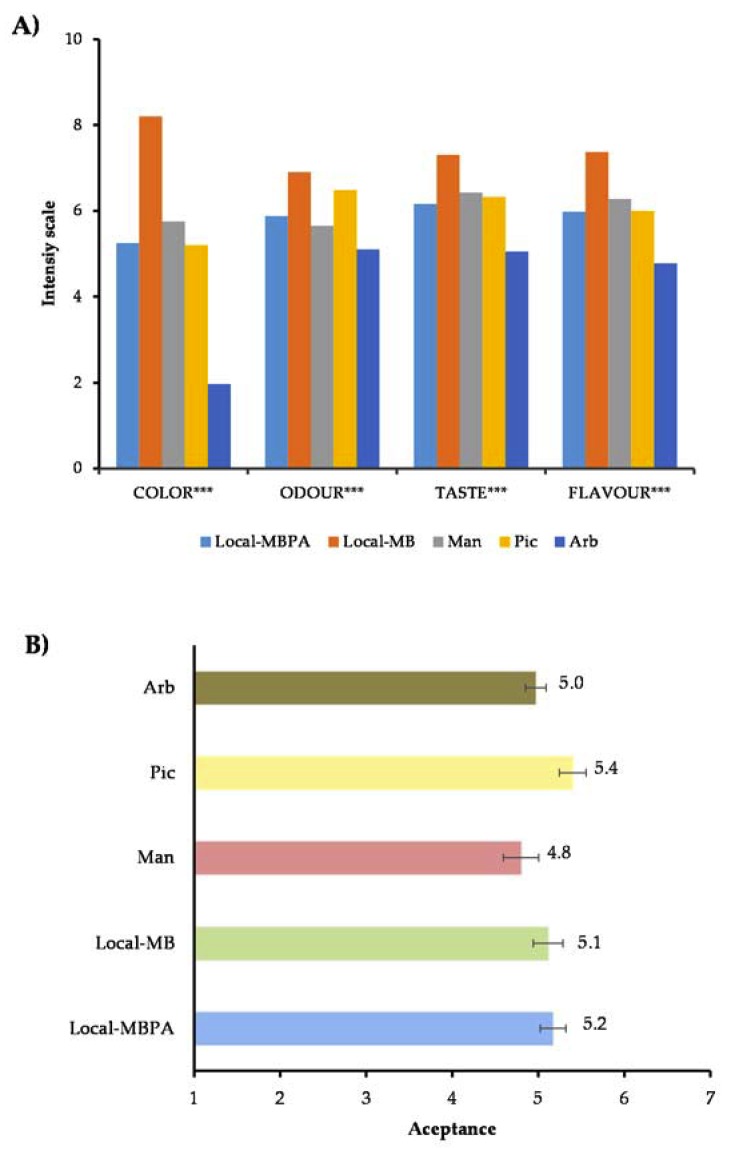
Intensity values obtained to sensorial attributes (**A**) and global acceptance of VOOs (**B**). *** (*P* ≤ 0.001); acceptance: 1 = dislike very much; 2 = dislike moderately; 3 = dislike slightly; 4 = neither like nor dislike; 5 = like slightly; 6 = like moderately; 7 = like very much). Local-MBPA (60% Mansa and Brava, 25% Picual, and 15% Arbequina); Local-MB (60% Mansa and 40% Brava); Man (100% Mansa cultivar); Pic (100% Picual cultivar); Arb (100% Arbequina cultivar).

The differences in physical and chemical parameters (fatty acids and volatile compounds) found in olive oils involve differences in their organoleptic properties which would have an effect on consumer acceptance. Sensory analysis is very important in the oil industry due to the quality and commercial oil categories being established by trained taste [13], but acceptance and preference evaluations are also very important to evaluate acceptability of new olive oils by consumers. The sensorial attributes considered to evaluate overall liking of olive oils by consumers were color, odor, taste, and flavor, and the intensity values of these sensorial attributes are showed in Figure 4A. The four studied attributes showed significant differences (*P* ≤ 0.001) between olive oils. The *Local*-MB oils presented the highest intensity values for the sensory attributes and *Arbequina* oils the lowest. *Local*-MBPC and *Picual* oils have obtained similar scores by color, taste, and flavor.

When the global acceptance was studied (Figure 4B), the consumers had a higher acceptance for *Picual* (5.4) following of *Local*-MBPA (5.2) and *Local*-MB (5.1) oils, but there were not significant differences (*P* ≥ 0.05).

The same way, total scores of preferences obtained to ranking test (number in brackets in Table 3) showed that *Picual*, following *Local*-MBPA and *Local*-MB oils, were the more chosen for the consumers and Friedman’s test indicated that there were significant differences (*P* ≤ 0.05) between olive oils (F_tes t_> F _= 0.05_). The results of the LSD test (Table 3) showed that the olive oils can be grouped in two groups well differentiated: one group composed by *Picual*, *Local*-MBPA, and *Local*-MB oils, and other group included *Arbequina* oils. *Mansa* oils presented significant differences with *Picual* oils.

**Figure 5:**
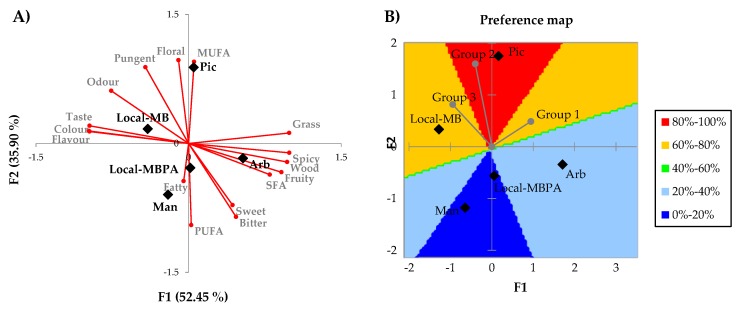
Attribute map (**A**) and external preference mapping and contour plot (**B**) of VOOs. Preference model Figure 1 and F2 of principal components analysis (PCA). Local-MBPA (60% Mansa and Brava, 25% Picual, and 15% Arbequina); Local-MB (60% Mansa and 40% Brava); Man (100% Mansa cultivar); Pic (100% Picual cultivar); Arb (100% Arbequina cultivar).

Preference mapping has been used extensively to describe the characteristics that contribute to consumers’ liking as well as which products they like most or least [25] and internal and external quality mapping has been applied to investigate sensory quality in EVOOs and uncover the positive and negative drivers of sensory quality as perceived by experts [12]. In this sense, external preference mapping (PREFMAP method [34]) was created to relate consumer acceptance, sensorial attributes, fatty acids, and volatile compounds of olive oil samples and to establish a quality-mapping of olive oils from the consumer viewpoints. The attribute map created previously at PREFMAP using principal components analysis (PCA) applied on instrumental and sensorial variables (Figure 5A) showed that the first two components accumulated 88.35% of total variation (F1 = 52.45%; F2 = 35.90%). The differences in the first dimension are more important than the differences in the second one. Reis et al. [35] considered that for success of the PCA, F1 and F2 have to accumulate a percentage of variance equal or greater than 70%. On the “heat map” (Figure 5B) it can be observed that all consumers (grouped in three groups with similar overall linking profiles using hierarchical cluster analysis (HCA)) had a preference above average in warm colors (yellow and red).

The resulting preference map indicated that vector model was the best, allowing representation of the observations as vectors. The longer the vector indicated better underlying model and the consumer’s linking increases, the further it moves away from the direction of the vector. The most appreciated samples were *Picual* and *Local*-MB oils which were positioned in the direction of the axis associated with high values for odor intensity and pungent and floral attributes (*Picual* oils) and high values for taste, color, and flavor intensity (*Local*-MB oils). As previously reported, bitter was not seen as a pleasant feature by consumers and they rejected this attribute which is considered a positive sensorial attribute of olive oil [15].

## 4. Conclusions

From the results, we concluded that the both Local oils can be classified as EVOOs, have a good oxidative stability, and are of good quality. Local-MB together with Picual oils show the best quality parameters in base of fatty acids composition. However, Local-MBPA oils register higher volatile compounds content than Local-MB oils probably due to enrichment given by Arbequina cultivar. On the other hand, Local-MB oils present the highest intensity values for color, odor, taste, and flavor, and Picual following of the both Local oils are the more chosen for the consumers. Finally, a quality-mapping of olive oils indicate that attributes more valued from the consumer viewpoints are high intensity for color, odor, taste, and flavor, and pungent and floral series. The bitter attribute is rejected by consumers which confirm the hypothesis that consumers are unfamiliar with this positive attribute of olive oils and it is necessary to provide more information about of the relation among bitter and pungent attributes and nutritional and healthy properties of olive oils.

## Figures and Tables

**Table 1:** Fatty acids (expressed as % of total fatty acids) of the studied virgin olive oils.

	*Local*-MBPA	*Local*-MB	Man	Pic	Arb	EVOO Reference *
C14:0	0.02 ± 0.00	0.01 ± 0.00	0.02 ± 0.00	0.01 ± 0.00	0.02 ± 0.00	≤0.03
C16:0	12.72 ± 0.63 ^b^	11.74 ± 0.19 ^c^	13.02 ± 0.02 ^b^	11.29 ± 0.10 ^c^	13.53 ± 0.11 ^a^	7.50–20.00
C16:1n-7	0.89 ± 0.009 ^c^	0.74 ± 0.04 ^d^	0.98 ± 0.01 ^b^	0.76 ± 0.01 ^d^	1.03 ± 0.01 ^a^	0.30–3.50
C17:0	0.14 ± 0.00 ^a^	0.09 ± 0.00 ^b^	0.14 ± 0.00 ^a^	0.05 ± 0.00 ^c^	0.14 ± 0.00 ^a^	≤0.40
C17:1n-7	0.28 ± 0.00 ^b^	0.20 ± 0.01 ^d^	0.36 ± 0.00 ^a^	0.10 ± 0.00 ^e^	0.26 ± 0.00 ^c^	≤0.60
C18:0	2.15 ± 0.008 ^c^	2.66 ± 0.03 ^b^	1.94 ± 0.01 ^d^	2.77 ± 0.00 ^a^	1.95 ± 0.00 ^d^	0.50–5.00
C18:1n-9	71.79 ± 1.74 ^bc^	73.99 ± 1.89 ^b^	67.07 ± 0.05 ^d^	77.72 ± 0.06 ^a^	69.60 ± 0.09 ^c^	55.00–83.00
C18:2n-6	7.92 ± 0.83 ^c^	6.80 ± 1.37 ^c^	11.75 ±0.00 ^a^	3.74 ± 0.01 ^d^	9.21 ± 0.00 ^b^	2.50–21.00
C18:3n-3	0.60 ± 0.01 ^d^	0.74 ± 0.04 ^b^	1.03 ± 0.00 ^a^	0.68 ± 0.00 ^c^	0.56 ± 0.00 ^e^	≤1.00
C20:0	0.43 ± 0.01 ^a^	0.41 ± 0.03 ^a^	0.35 ± 0.01 ^b^	0.39 ± 0.02 ^a^	0.41 ± 0.00 ^a^	≤0.60
C20:1n-9	0.30 ± 0.01 ^a^	0.27 ± 0.01 ^b^	0.25 ± 0.00 ^c^	0.23 ± 0.00 ^d^	0.30 ± 0.00 ^a^	≤0.50
C22:0	0.14 ± 0.00 ^a^	0.12 ± 0.00 ^b^	0.10 ± 0.00 ^c^	0.11 ± 0.00 ^c^	0.13 ± 0.00 ^ab^	≤0.20
C24:0	0.08 ± 0.02 ^a^	0.06 ± 0.01 ^a^	0.04 ± 0.00 ^b^	0.06 ± 0.00 ^a^	0.07 ± 0.00 ^a^	≤0.20
*t*-oleic isomers	n.d.	n.d.	n.d.	n.d.	n.d.	≤0.05
*t*-linoleic + *t*-linolenic	n.d.	n.d.	n.d.	n.d.	n.d.	≤0.05

* Legally establish ranges (European Union Commission, [16,17]). Values are mean ± standard deviation (*n* = 3). n.d. = not detected (<LOD). ^a–d^ Mean values in the same row with different letters indicate significant differences (*P* < 0.05). Local-MBPA (60% Mansa and Brava, 25% Picual, and 15% Arbequina); Local-MB (60% Mansa and 40% Brava); Man (100% Mansa cultivar); Pic (100% Picual cultivar); Arb (100% Arbequina cultivar).

**Table 2:** Volatile compounds found in studied VOOs and their aromatic characteristics.

Volatile Compound	*m*/*z*	Sensory Descriptor	Odorant Series	Mouth Sensation	Reference
Ethyl formate	74	ethereal, green, rose	Floral		[32]
2-Methylpropanal	72	pungent, nutty	Spicy	Pungent	[22]
2-Butanone	72	ethereal, fragrant, pleasant, fruity, mushroom	Fruity, Spicy		[33]
2-Methyl-3-Buten-2-ol	71	herbal, mushroom	Grass, Spicy		[32]
3-Methylbutanal	58	malty, fruit, acorn-like	Fruity		[33]
2-Methylbutanal	58	malty	Fruity		[33]
1-Penten-3-one	55	green, bitter, pungent, mustard	Grass	Pungent	[11]
2-Pentanone	86	ethereal, butter, spiced	Spicy, Fatty		[32]
3-Pentanone	86	olive fruit, sweet	Fruity	Sweet	[11]
Acetoin	45	buttery, sweet	Fatty	Sweet	[32]
3-Methylbutanol	70	whiskey, woody, sweet	Wood	Sweet	[33]
2-Methylbutanol	56	pungent		Pungent	[33]
Octane	85	Green, minty, herbaceous (rosemary), lime, lemon, woody	Grass, Wood, Spicy		[32]
*cis*-2-Penten-1-ol	57	olive fruit, sweet, banana	Fruity	Sweet	[11]
Hexanal	56	grass, green apple	Grass		[11]
1-Methoxyhexane	45	herbal, floral, lavender	Floral		[32]
Ethyl 2-methylbutirate	102	fruity	Fruity		[33]
*trans*-2-Hexenal	98	grass, apple-like, bitter, bitter almond, green	Grass, Fruity	Bitter	[11]
*cis*-3-Hexen-1-ol	67	leaf, apple, bitter, green grass, herbal	Grass, Fruity	Bitter	[11]
1-Hexanol	56	olive fruit, banana, green grass	Grass, Fruity		[11]
Dimethyl sulfide	62	cabbage, garlic, onion	Spicy		[32]
Heptanal	70	wood, oily, green plant	Wood, Fatty, Grass		[33]
*trans*, *trans*-2,4-Hexadienal	81	green, sweet, fruit, citrus, waxy	Grass, Fruity		[32]
Methoxymethylbenzene	122	ethereal, green, hyacinth, floral	Floral		[32]
*cis*-3-Hexenyl acetate	82	green, fruity, banana	Grass, Fruity		[11]
Hexyl acetate	61	grass, olive fruit, sweet	Grass, Fruity	Sweet	[11]
b-Ocimene	93	sweet, green	Grass	Sweet	[32]
3-Carene	56	pungent odor, fir needles, mushroom	Grass	Pungent	[32]
Benzeneacetaldehyde	91	acorn, pungent	Grass	Pungent	[22]
Nonanal	98	citrus-like, waxy, pungent	Fatty, Floral, Grass	Pungent	[22]
Phenylethyl Alcohol	91	floral, sweet	Floral	Sweet	[32]
a-Copaene	161	woody, spicy, honey	Wood, Spicy		[32]

*m*/*z*: quantifier ion used in the GC-MS analysis.

**Table 3:** Total preference values and LSD results obtained from the studied VOOs.

Sample Most Favorite				Sample Least Favorite
Pic (246)	*Local*-MBPA (220)	*Local*-MB (216)		
	*Local*-MBPA (220)	*Local*-MB (216)	Man (190)	
			Man (190)	Arb (178)

Samples in the same row not have significant differences (*P* > 0.05) and samples in different row show significant differences (*P* ≤ 0.05). Numbers in brackets are ∑ score. Local-MBPA (60% Mansa and Brava, 25% Picual, and 15% Arbequina); Local-MB (60% Mansa and 40% Brava); Man (100% Mansa cultivar); Pic (100% Picual cultivar); Arb (100% Arbequina cultivar).
